# The close proximity of threat: altered distance perception in the anticipation of pain

**DOI:** 10.3389/fpsyg.2015.00626

**Published:** 2015-05-13

**Authors:** Abby Tabor, Mark J. Catley, Simon C. Gandevia, Michael A. Thacker, Charles Spence, G. L. Moseley

**Affiliations:** ^1^Sansom Institute for Health Research, University of South AustraliaAdelaide, SA, Australia; ^2^School of Biomedical Sciences, Center of Human and Aerospace Physiological Sciences and Pain Research Section, Neuroimaging, Institute of Psychiatry, King’s College LondonLondon, UK; ^3^Neuroscience Research Australia – University of New South WalesSydney, NSW, Australia; ^4^Department of Experimental Psychology, University of OxfordOxford, UK

**Keywords:** perceptual inference, pain, neuroeconomics, Bayesian decision-making, persipersonal space

## Abstract

Pain is an experience that powerfully influences the way we interact with our environment. What is less clear is the influence that pain has on the way we perceive our environment. We investigated the effect that the anticipation of experimental pain (THREAT) and its relief (RELIEF) has on the visual perception of space. Eighteen (11F) healthy volunteers estimated the distance to alternating THREAT and RELIEF stimuli that were placed within reachable space. The results determined that the estimated distance to the THREAT stimulus was significantly underestimated in comparison to the RELIEF stimulus. We conclude that pain-evoking stimuli are perceived as closer to the body than otherwise identical pain-relieving stimuli, an important consideration when applied to our decisions and behaviors in relation to the experience of pain.

## Introduction

The world we perceive is constructed on the basis of previous experiences, current information, and predicted outcomes (Yuille and Kersten, 2006; Chater et al., 2010). Such perceptual constructs are individually efficient, but also subject to bias (Helmholtz, 1866; Gregory, 1997). Our perceptions are thought to reflect the utility of the self within an environment; a so-called ‘neuroeconomic’ vantage point, from which we interpret incoming information in line with the most likely and ‘cost-effective’ hypothesized outcome (Körding et al., 2004; Gershman and Daw, 2012).

Pain is a high cost experience, tied to the probability that the body is under threat. In contrast, relief from pain can be considered a beneficial experience, tied to the removal of that threat. Both pain and relief powerfully compel us to behave in a way that promotes our protection and recovery (Bentham, 1879). These behaviors are readily observed in both acute and persistent pain and have been interpreted as reflecting mechanisms to avoid or reduce pain or (re) injury (Vlaeyen and Linton, 2012). However, this process is unlikely to be in one direction, with the way in which we construct our perceptual inferences both influencing, and being influenced by, our behavior (Noë, 2002; Kersten et al., 2004).

Conventionally, pain research focuses on behavior, subject affect, physiology as well as cognition (Mcmahon et al., 2013). Yet, as an experience that affects the way we interact with our environment, little is known about the influence that pain has on the way we construct the perceptions of our environment. This line of investigation sits amidst an ongoing debate as to the true extent of ‘top–down’ effects on perception (Cecchi, 2014). A number of studies have demonstrated that perceptions are altered in a way that reflects the physiological and psychological state of an observer in their environment (Steffanucci and Proffitt, 2009; Proffitt, 2013). Indeed, under this embodied inference approach to perception, it has been proposed that threatening objects are perceived as closer than equivalent controls (Cole et al., 2013), similarly it has been demonstrated that objects that we desire are perceived as closer than neutral objects (Balcetis and Dunning, 2010; Alter and Balcetis, 2011; Takahashi et al., 2013). However, astute methodological counters have proposed that the actual biases may arise as the result of response and memory effects (Firestone, 2013; Firestone and Scholl, 2014). Thus, minimizing the opportunity for such biases to occur is particularly important to gain further insight into the cognitive penetrability, or lack there of, of perception.

The experience of pain provides a unique vantage point from which to explore the extent of ‘top–down’ influences on perceptual inference, incorporating the powerful behavioral drivers of threat and relief. Drawing on previous findings (Balcetis and Dunning, 2010; Alter and Balcetis, 2011; Cole et al., 2013; Tabor et al., 2013), objects associated with these two opposing drivers are seen as closer in comparison to control. Yet, what has not been investigated is how these two influences on perception interact with one another, specifically the effect of viewing a stimulus associated with threat in comparison to a stimulus that offers relief.

This line of enquiry is a particularly important consideration in relation to pain seen in clinical practice, as it considers the possibility that the experience of pain, which is known to profoundly influence the way one acts in their environment, could influence the way one visually perceives their environment, thereby introducing a circular causality effect previously not considered.

Adopting this direction, the present study looked to gain insight into the way in which perceptions are constructed in relation to pain and relief and thus providing an alternative perspective for the investigation and potential treatment of pain. We used an experimental pain paradigm to explore whether the perceived distance to specific stimuli is modulated by the relevance of those stimuli to the experiences of the threat of pain and the relief of pain. Based on the biological relevance for actively avoiding interactions with painful stimuli, as well as the increasing aversion related to future pain (Story et al., 2013), we hypothesized that pain-evoking stimuli would be perceived as closer to the body than otherwise identical pain-relieving stimuli.

### Materials and Methods

#### Participants

A convenience sample of 18 (11F; mean age: 21 years; SD ± 2) healthy volunteers participated. The sample size was informed by previous findings in this field and determined using G^∗^Power (Faul et al., 2007), specifying 0.8 power and a medium effect size, with the α set at 0.05; data collection stopped when this number was satisfied. All volunteers had participated in a previous experiment (Tabor et al., 2013), but had not been exposed to the THREAT condition used here; the two experiments were separated by a 15 min interval. Participants were excluded if they had a history of pain lasting more than 3 months or had pain at the time of the experiment. All participants reported no abnormal neurological symptoms and provided written informed consent. The experimental protocol was approved by the institutional ethics committee and conformed to the World Medical Association (2013) and the local national code for the Responsible Conduct of Research.

#### Stimulus Apparatus

A noxious heat stimulus was delivered using a Medoc system (Ramat Yishai, Israel; http://www.medoc-web.com) with a Pathway ATS thermode, driven by TSA-2001 software. Individual heat pain thresholds were established using the threshold by limits method (Yarnitsky and Sprecher, 1994). A RED and a BLUE wireless computer mouse were used as the switch in both conditions. In the control condition, each switch was placed alternately along the midline of the table, associated with no other stimulus. In the test condition, the red switch and the blue switch were presented in alternate fashion, but the red switch when activated produced a noxious heat stimulus (THREAT) whereas activating the blue switch resulted in the cessation of the noxious heat stimulus (RELIEF). The stimulus was either delivered (in the THREAT condition) or removed (in the RELIEF condition) immediately on pressing the relevant switch.

#### Assessments

Prior to the test condition (see Test Phase), the heat pain threshold of each participant was determined. The thermode (3 cm × 3 cm) was placed on the back of the participant’s non-dominant hand, with the standard control button held in their dominant hand. We informed the participant that the temperature of the thermode would steadily increase (at 2°C per second) and that when the stimulus first became painful, they were to click the control switch, which would return the temperature of the thermode to the baseline temperature (30°C) at a rate of 8°C per second. This process was repeated four times. Pain thresholds were calculated by averaging the outcomes of trials 2–4. Trial 1 was discarded to allow for habituation (Becerra et al., 1999).

#### Distance Estimates

Participants were shown a one-centimeter measure and a meter rule prior to testing for reference. Participants were instructed to verbalize a distance estimate (to the nearest centimeter) from their non-dominant hand to the base of the switch that was placed at varying distances, within an arm’s reach, in front of them.

#### Experimental Protocol

We used a repeated-measures design, comprising of two phases: a control phase and a test phase, the test phase involved two active experimental conditions. The nature of the phase was described before it commenced and the start of each phase was preceded with an example run of the condition so as to actively demonstrate the nature of the task.

In each phase the participants were instructed to place both hands (dominant hand resting on non-dominant hand) behind a line drawn 5 cm from the nearside edge of a large, blank table and to bring their body abreast with the edge of the table and their hands; they were then asked to close their eyes. The investigator placed the colored switch along the midline of the table, randomly at one of five set distances: 25, 30, 35, 40 and 45 cm; live video feedback via a ceiling mounted webcam linked to a laptop was used to guide accurate placement. The participant was blinded to the pre-determined distances and the visual feedback throughout the trial. In each condition of the phases, the switch was placed 10 times, twice at each distance and remained at that distance for a single trial until the participant had verbalized their distance estimate, actively clicked the switch and closed their eyes, which marked the end of each trial. The participant was allocated 3 s to view the distance before being asked by the experimenter to provide a prompt distance estimate, each trial lasted no longer than 8 s.

##### Control Phase

The control phase was performed first in all participants so that no previously encountered stimulus was associated with the target. Participants were informed that they were required to estimate the distance to a switch placed in front of them; it was outlined that they would view alternating red and blue switches, both of which were inactive and not associated with an additional stimulus. On each presentation of the switch, participants were verbally prompted to “estimate the distance to the inactive switch, to the nearest centimeter,” once they had verbally given their estimate, the participant reached and clicked the switch.

##### Test Phase

The test phase began with the application of the thermode device to the back of the participant’s non-dominant hand, followed by the completion of the threshold protocol outlined in Section “Assessments.” The phase involved the same alternating red and blue switches, but this time the participants were informed that they were activated. It was clearly outlined that the red switch, when clicked by the participant would activate the thermode on the back of their non-dominant hand, delivering a noxious heat stimulus set to their pain threshold. In contrast, the blue switch when clicked would deactivate the thermode and offer RELIEF from a noxious heat stimulus.

###### ‘THREAT’ condition

The experimenter prompted the participant to open their eyes, marking the beginning of the trial. On viewing the THREAT switch, participants were allocated 3 s before being verbally prompted to “estimate the distance to the switch that will activate a noxious stimulus, to the nearest centimeter.” Following their distance estimation the participant was required to reach and click the switch, which activated the thermode and a noxious stimulus was delivered. This stimulation lasted for 5 s, after which time the switch was deactivated by the experimenter via laptop control of the thermode. The participant indicated the end of the trial by returning their hand to behind the line and closing their eyes.

###### ‘RELIEF’ condition

The experimenter instigated each RELIEF trial by activating the thermode and the delivery of a noxious stimulus via laptop control of the thermode. The RELIEF trial would begin when the participant, who had their eyes closed following the completion of the preceding THREAT trial, opened their eyes; the participants were instructed to only open their eyes when the stimulus applied to the back of their hand had become painful. After viewing the switch for 3 s, throughout which time they were experiencing noxious stimulation, the participant was prompted to “estimate the distance to the switch that will deactivate the noxious stimulus, to the nearest centimeter.” Following the distance estimation the participant was required to reach and click the switch to relieve them of the noxious stimulation. The participant returning their hand to behind the line and closing their eyes marked the completion of a trial and readiness for the next THREAT trial. This alternation occurred throughout the test phase until each predetermined distance was estimated twice.

#### Statistical Analysis

All analyses were conducted using PASW Statistics (v18.0.0, IBM Corporation, Armonk, NY, USA).

To test whether differences existed between the conditions a repeated measures four [Control red, Control blue, Test red (THREAT), Test blue (RELIEF)] × five (Distance Estimate – five levels) ANOVA was undertaken. To test the primary hypothesis, we undertook a two (Condition – THREAT or RELIEF) × five (Distance – five levels) ANOVA. If such an effect was evident, we replicated the analysis on the control experiment data. Additionally, if a significant interaction was detected, suitable *post hoc* tests were carried out.

If the data did not meet the assumptions of parametric statistics, the equivalent non-parametric tests were used. Significance of all statistical tests was set at α = 0.05. In the case that sphericity was violated, the greenhouse-geisser correction was utilized.

### Results

The results of the first ANOVA showed that participants were significantly different in their estimation of distance between the conditions [*F*(3,51) = 4.376; *p* = 0.024; *n*^2^ = 0.2]. The results also demonstrated a significant effect of distance [*F*(4,68) = 3.393; *p* = 0.04; *n*^2^ = 0.17]. In addition, the repeated measures ANOVA revealed a significant Condition^∗^Distance interaction [*F*(12,204) = 2.552; *p* = 0.42; *n*^2^ = 0.13]. *Post hoc* tests, using a Bonferroni correction, revealed that only distance estimations made in the THREAT condition compared with distance estimates made within the RELIEF condition were found to be significantly different (*p* = 0.04; see Supplementary Material for all pairwise comparisons). That is, there was a significant effect of Condition, with an underestimation of distance in the THREAT condition as compared to the RELIEF condition, irrespective of the Distance level.

**FIGURE 1:**
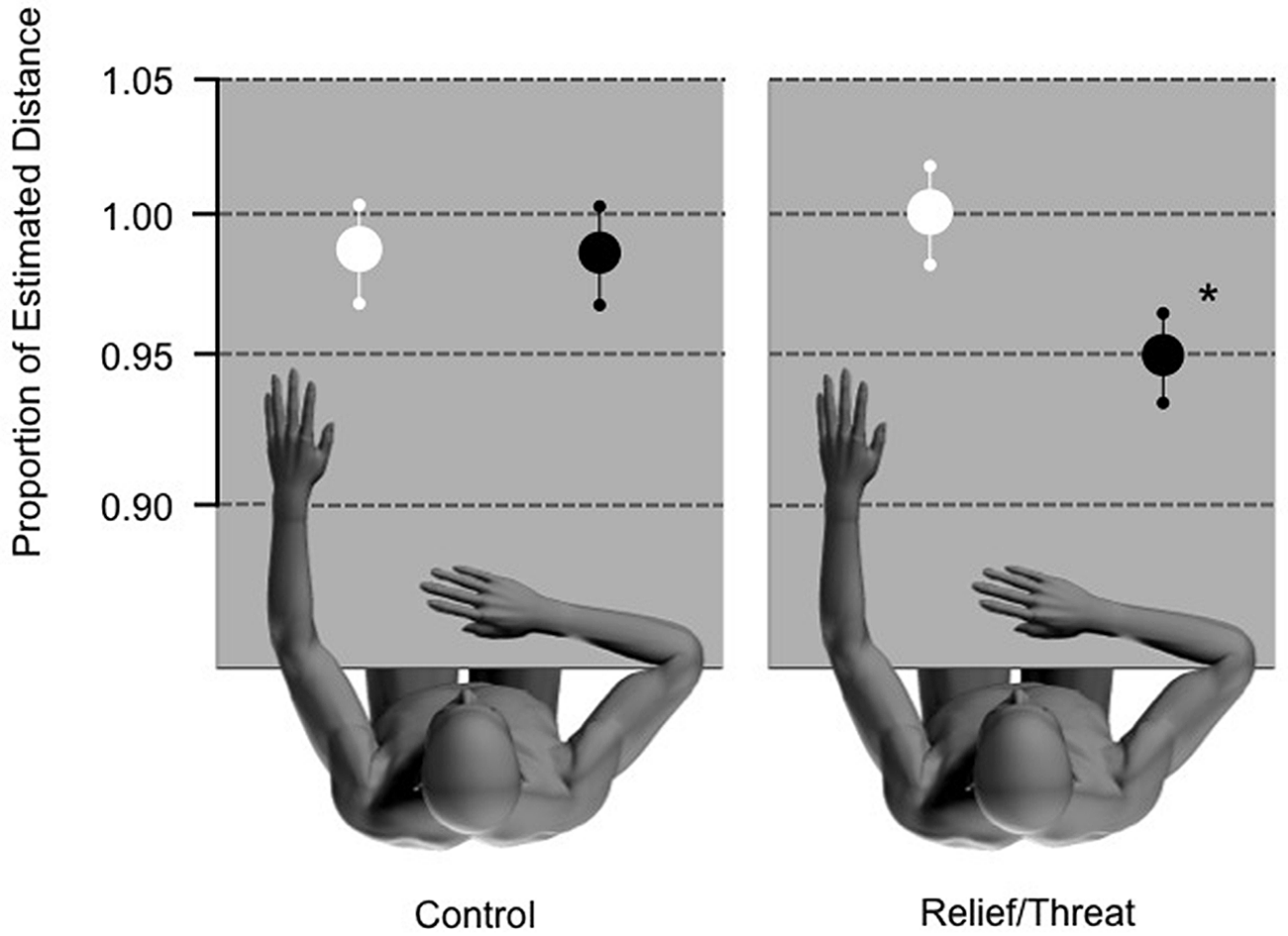
**Control and test conditions**. ^∗^Significant underestimation of THREAT (black circles) stimulus as compared to RELIEF (white circles) stimulus (*p* = 0.007; *n*^2^ = 0.6). Ninety-five percentage confidence intervals are presented for each condition.

Testing the primary hypothesis, the second ANOVA confirmed that participants significantly underestimated the distance to the THREAT switch in comparison to the RELIEF switch, with a significant effect of condition [*F*(1,17) = 9.543; *p* = 0.007; *n*^2^ = 0.6; see **Figure 1**] in the test stage. The mean (±SD) estimate as a proportion of the actual distance was 0.952 (±0.147) for the THREAT switch and 1.008 (±0.147) for the RELIEF switch (see **Table 1** for all proportional distances). There was no significant effect of distance (*p* = 0.641) and no significant condition^∗^distance interaction (*p* = 0.308).

**Table 1:** Proportional distance estimations.

	25 cm	30 cm	35 cm	40 cm	45 cm
Control red	0.92	0.98	1.01	1.03	1.01
Control blue	0.91	0.96	1.03	0.99	1.00
Threat red	0.92	0.96	0.94	0.97	0.97
Relief blue	1.03	1.02	0.99	0.99	1.01

*Participant estimations, represented as proportions of actual distances, are presented for each condition and for each distance*.

As our results demonstrated that there was a significant effect in the test stage, we wanted to confirm that such an effect was not simply due to the alternating color of the switch but rather the association of the switch with either relief from pain or threat of pain. The control stage used the same alternate computer mouse presentation as in the test condition, without the associated THREAT or RELIEF stimuli. We observed no difference between the estimates of distance in the control conditions (*p* = 0.171). As such, the effect that we observed in the experiment was not imparted by the color of the switch, but rather the association of the switch color and it’s relevance to THREAT or RELIEF. In addition, we found a significant effect of distance [*F*(3,68) = 6.367; *p* = 0.003; *n*^2^ = 0.27], however, no significant interaction condition^∗^distance (*p* = 0.294).

### Discussion

We hypothesized that pain-evoking stimuli would be perceived as closer to the body than otherwise identical pain-relieving stimuli; our results support this hypothesis. This result is consistent with the idea that our perceptions are inferential and reflect the context in which the information is integrated (Yuille and Kersten, 2006). Moreover, it suggests that a previously demonstrated perceptual bias, an underestimation of distance when observing a relieving stimulus in isolation (Balcetis and Dunning, 2010; Tabor et al., 2013), can be over-ridden when presented with an opposing stimulus. One explanation for this overriding effect could be the value associated with the anticipation of an unknown threatening stimulus as contrasted with the value associated with the relief from a known threatening stimulus. This is consistent with the idea that the dread and aversion attributed to a noxious stimulus increases with increasing time delay (Story et al., 2013). In the pain-relieving condition the participant is already experiencing the noxious stimulus when estimating the distance to a target, whereas in the pain-evoking condition the noxious stimulus was delayed and therefore anticipated following the participant’s distance estimate.

Clinical data demonstrate that people with persistent pain adopt protective responses in situations that are not actually dangerous. That these responses may reflect altered perceptual inference has been mooted (Butler and Moseley, 2003), but, until now, empirical support for this possibility has been lacking. The present findings suggest that when people anticipate pain, or perceive a stimulus in their environment as threatening, their perceptual appraisal of that environment may be altered. Clearly, we do not experience our environment in isolation. Rather, we continually update, making inferences based on what has occurred prior to the present and in the shadow of what we anticipate in the future (Dalgleish et al., 2009). The current findings may reflect an alternating perceptual bias evoked by the comparison of opposing, highly salient stimuli. Investigation of the effect of a threatening stimulus when seen in isolation as well as in association with a competitive stimulus seems warranted (Leknes et al., 2011).

Our results are in keeping with the recent discovery of ‘defensive personal space,’ whereby the defensive hand blink reflex, previously held to be under automatic control, shows clear modulation according to (i) the distance between the hand and the face and (ii) the presence or not of a physical barrier between them (Sambo et al., 2012). This implies that bottom–up and top–down mechanisms interact to inform our perception of self and the surrounding environment, involving the physiological regulation of our body, inseparable from the space it inhabits (Tsakiris and Haggard, 2005; Moseley et al., 2012; Wiech and Tracey, 2013). The current results further this proposal showing that the very location of a threatening object presented in peripersonal space is altered in relation to the anticipation of the threat, emphasizing the circular causality of these relationships.

The proposal that ‘top–down’ effects influence perceptual inference is currently hotly debated across psychology, neuroscience and philosophical realms (Firestone, 2013; Firestone and Scholl, 2014; Shea, 2014). Pain is widely accepted as an experience that is modulated by ‘top–down’ effects, as well as incoming sensory information. The present study looks to extend this notion to the impact that the presence and absence of pain itself has on visual inferences that we make. This consideration is in its infancy, however, the hypothesis at large may serve to shed light on pain-related decision-making both in research and in clinical practice; where, in the absence of knowledge about the inferences the individual is making about their environment, overt behavior may be framed as irrational.

Interpretation of this work should consider that a presumption was made about the meaning of the stimuli for participants, specifically the experiences of THREAT and RELIEF. Although previous work underpins that presumption (Moseley and Arntz, 2007; Tabor et al., 2013), verifying the actual experience of the participant would have offered clear confirmation; future work would benefit from the addition of such reports as well as physiological and psychological measures to better establish the experience of the participants. The color of the switches were chosen deliberately, therefore a progression of the current study could be to introduce a neutral colored switch or a condition in which the colors are reversed to further investigate the nature of the effect. Also, participants were naïve to the hypothesis of the study and emphasis was placed on estimate accuracy in an attempt to minimize response bias. Finally, our participants had previously been in a study that involved relief stimuli and the order of these experiments were not counterbalanced, which may have introduced a prior expectancy and altered their performance here. Importantly, however, the direction of our observed bias in the present study overrides that of the previous study, which strongly suggests against a prior expectancy and response bias.

### Conclusion

Pain-evoking stimuli are perceived as closer to the body than otherwise identical pain-relieving stimuli. This finding supports the notion that our perceptions are inferences, constructed in relation to our prior encounters, and relevant incoming information.

### Author Contributions

AT: Primary author, development of idea, manuscript preparation, data collection, analysis, and interpretation. MC: Development of idea, on-going manuscript editing, data collection. SG: Initial concept, development of idea, manuscript editing. MT: Development of idea, manuscript editing. CS: Development of idea, manuscript editing. GM: Initial concept, development of idea, data analysis, and interpretation, on-going manuscript editing.

## Conflict of Interest Statement

The authors declare that the research was conducted in the absence of any commercial or financial relationships that could be construed as a potential conflict of interest.
