# Supplementary material for: The close proximity of threat: altered distance perception in the anticipation of pain
Source: Front Psychol. 2015 May 13;6:626. doi: 10.3389/fpsyg.2015.00626 (PMC4429615; doi:10.3389/fpsyg.2015.00626)
Supplement: Supplementary file 1 [file Table_1.PDF]

*Supplementary Material*

# The close proximity of threat: Altered distance perception in the anticipation of pain

Abby Tabor[1,3], Mark J Catley[1], Simon Gandevia[2], Michael A Thacker,[1,3] Charles Spence[4] & G Lorimer Moseley[1, 2 *]

[1] Sansom Institute for Health Research, University of South Australia, Australia.

[2] Neuroscience Research Australia, Sydney and University of New South Wales, Australia.

[3] School of Biomedical Sciences, Centre of Human and Aerospace Physiological Sciences and Pain Research Section, Neuroimaging. Institute of Psychiatry, King's College London, United Kingdom.

[4] Department of Experimental Psychology, University of Oxford, Oxford, United Kingdom.

* Corresponding Author:
G. Lorimer Moseley
University of South Australia
GPO Box 2473
Adelaide SA 5001, Australia
Telephone: +61 8 830 21416
Facsimile: +61 8 830 22853
email: lorimer.moseley@gmail.com
URL: www.bodyinmind.org

Table 1. Post hoc analyses ANOVA 1 applying Bonferroni correction, pairwise comparisons between Conditions.

| **Condition** | | Mean Difference | *Significance* |
|---|---|---|---|
| **Control Red** | Control Blue | 1.09 | 1.00 |
| | THREAT | 3.57 | 0.06 |
| | RELIEF | -1.92 | 1.00 |
| **Control Blue** | Control Red | -1.09 | 1.00 |
| | THREAT | 2.47 | 0.50 |
| | RELIEF | -3.01 | 1.00 |
| **THREAT** | Control RED | -3.57 | 0.06 |
| | Control Blue | -2.47 | 0.50 |
| | RELIEF | **-5.48*** | **0.04** |
| **RELIEF** | Control Red | 1.92 | 1.00 |
| | Control Blue | 3.01 | 1.00 |
| | THREAT | **5.48*** | **0.04** |

Table 2. Post hoc analyses ANOVA 1 applying Bonferroni correction pairwise comparisons between Distance levels

| **Distance** | | Mean Difference | *Significance* |
|---|---|---|---|
| **Level 1: 25cm** | 30cm | -3.67 | 0.12 |
| | 35cm | -5.08 | 0.11 |
| | 40cm | -5.12 | 0.14 |
| | 45cm | -5.90 | 0.31 |
| **Level 2: 30cm** | 25cm | 3.67 | 0.12 |
| | 35cm | -1.41 | 1.00 |
| | 40cm | -1.45 | 1.00 |
| | 45cm | -2.23 | 1.00 |
| **Level 3: 35cm** | 25cm | 5.08 | 0.11 |
| | 30cm | 1.41 | 1.00 |
| | 40cm | -0.04 | 1.00 |
| | 45cm | -0.82 | 1.00 |
| **Level 4: 40cm** | 25cm | 5.12 | 0.14 |
| | 30cm | 1.45 | 1.00 |
| | 35cm | 0.04 | 1.00 |
| | 45cm | -0.78 | 1.00 |
| **Level 5: 45cm** | 25cm | 5.90 | 0.31 |
| | 30cm | 2.23 | 1.00 |
| | 35cm | 0.82 | 1.00 |
| | 40cm | 0.78 | 1.00 |
